# Transplantation of human embryonic stem cells onto a partially wounded human cornea *in vitro*

**DOI:** 10.1111/j.1755-3768.2011.02358.x

**Published:** 2013-03

**Authors:** Charles Hanson, Thorir Hardarson, Catharina Ellerström, Markus Nordberg, Gunilla Caisander, Mahendra Rao, Johan Hyllner, Ulf Stenevi

**Affiliations:** 1 Unit of Clinical Sciences, Department of Obstetrics and Gynecology, Sahlgrenska University Hospital, University of GothenburgGöteborg, Sweden; 2 Fertilitetscentrum, Carlanderska HospitalGothenburg, Sweden; 3 Cellartis ABGöteborg, Sweden; 4 Life TechnologiesFrederick, MD, USA; 5 Department of Ophthalmology, Sahlgrenska University Hospital/MölndalMölndal, Sweden

**Keywords:** CK3, cornea, embryonic stem cells, epithelia, PAX6

## Abstract

**Purpose:**

The aim of this study was to investigate whether cells originating from human embryonic stem cells (hESCs) could be successfully transplanted onto a partially wounded human cornea. A second aim was to study the ability of the transplanted cells to differentiate into corneal epithelial-like cells.

**Methods:**

Spontaneously, differentiated hESCs were transplanted onto a human corneal button (without limbus) with the epithelial layer partially removed. The cells were cultured on Bowman’s membrane for up to 9 days, and the culture dynamics documented in a time-lapse system. As the transplanted cells originated from a genetically engineered hESC line, they all expressed green fluorescent protein, which facilitated their identification during the culture experiments, tissue preparation and analysis. To detect any differentiation into human corneal epithelial-like cells, we analysed the transplanted cells by immunohistochemistry using antibodies specific for CK3, CK15 and PAX6.

**Results:**

The transplanted cells established and expanded on Bowman’s membrane, forming a 1–4 cell layer surrounded by host corneal epithelial cells. Expression of the corneal marker PAX6 appeared 3 days after transplantation, and after 6 days, the cells were expressing both PAX6 and CK3.

**Conclusion:**

This shows that it is possible to transplant cells originating from hESCs onto Bowman’s membrane with the epithelial layer partially removed and to get these cells to establish, grow and differentiate into corneal epithelial-like cells *in vitro*.

## Introduction

A healthy epithelium is a prerequisite for the cornea to remain transparent. Corneal epithelial cells are generated from stem cells located mainly in the limbus area (Pellegrini et al. 1999; Dua & Azuara-Blanco 2000; Dua et al. 2005). In the absence of stem cells (aniridia) or after the destruction of the limbal stem cells (chemical injury), corneal epithelial cells are replaced by conjunctival epithelium, leading to corneal clouding and severely reduced vision. The treatment that can be offered in unilateral chemical injury is the transplantation of adult corneal stem cells from the patient’s other healthy eye. In bilateral cases and aniridia, more complex treatment modalities are possible, such as keratoprosthesis, keratolimbal stem cell transplantation, or homologous penetrating central limbo-keratoplasty (Reinhardt et al. 1999). In most cases, a donated cornea is required for surgical intervention, and in keratolimbal stem cell transplantation, two donor corneas are needed. However, there is a shortage of donated tissues and organs, not only in Sweden, but also worldwide. Furthermore, in many countries, religious and/or political views impede the use of donated material.

Human embryonic stem cells (hESCs) are cells originating from the inner cell mass of the blastocyst. These cells are pluripotent, meaning that they have the potential to differentiate into all cell types found in the human body (Heins et al. 2004). Human embryonic stem cells may preserve their pluripotency during long-term culture, and hence may be useful in several scientific and clinical applications (Mountford 2008).

If hESCs can be made to differentiate into corneal epithelial cells, the supply of cells for transplantation will be unlimited, thus decreasing the need for corneal transplants in the future. Therefore, as a first step, the aim of this study was to investigate whether cultured hESCs could be successfully transplanted onto a damaged human cornea. A second aim was to study whether the transplanted cells were prone to differentiate into human corneal epithelial-like cells using a conditioned milieu.

## Material and Methods

### Human corneas

Corneal tissue was obtained from patients who underwent primary penetrating keratoplasty for keratoconus or corneal decompensation. The removed corneal button, 7.5–8 mm in diameter, was kept in minimal essential medium (Invitrogen, Paisley, UK) until the surgery was completed, and then transferred to the laboratory for further processing. Prior to each experiment, the corneal epithelium was partially removed using a scalpel, before the stem cells were applied to the exposed Bowman’s membrane.

### Human ES cells

Human embryonic stem cells line SA121 (Cellartis, Goteborg, Sweden, http://www.Cellartis.com) (Heins et al. 2004) was genetically modified as described by Thyagarajan et al. (2008), to constitutively express green fluorescent protein (GFP) under the EF1-α promoter. The genetically modified hESC line, now denoted #277.1, had previously been characterized to confirm that the cells had remained pluripotent and diploid normal through the transfection procedures (data not shown). This clone was used to facilitate the identification of the transplanted cells in the time-lapse experiment and the immuno-histochemistry analysis. Initially, the hESCs were cultured in Cellartis DEF-Culture System (DEF-CS; Cellartis), which include neither feeder cells nor any type of membrane. Before transplantation, the homogenously undifferentiated hESCs were allowed to initiate differentiation. The cells were then cultured in VitroHES (Vitrolife, Göteborg, Sweden), 5% FBS (Gibco, Paisley, UK) and 10 μg/ml Hygromycin (Invitrogen, Carlsbad, CA, USA) for up to 22 days, hereafter referred to as the ‘preculture period’. On the day of the transplantation, the differentiation medium was removed and the cells were rinsed twice with 1× PBS ^+Ca/Mg+^ before transplantation medium was added [VitroHES, 5% FBS and 5 μm of ROCK Inhibitor (Y-27632; Sigma-Aldrich, Stockholm, Sweden)].

### Transplantation

The precultured cells (derived from hESC) were cut into pieces (2 × 2 mm) and placed in direct contact with Bowman’s membrane, after stripping most of the recipient epithelial cells.

### Time-lapse

After the preculture period, the GFP-expressing cells were cultured for up to 9 days together with the corneal tissue. During this period, the interaction between the transplanted cells and the recipient cells was observed through time-lapse photography. The time-lapse system has been previously described (Hardarson et al. 2004); in brief, the system was based around a mini incubator placed on an inverted microscope, connected to both premixed gas (6% CO_2_, 5% O_2_, and 89% N_2_) and an external heat source, creating physiological culture conditions. Pictures were taken at 5-min intervals during the entire culture period and used to create a time-lapse film recorded on a computer. The medium was changed regularly, at 2- to 3-day intervals throughout.

### Fixation and paraffin embedding

The cornea was removed from the time-lapse system and fixed in 4% formaldehyde for 24 hr, then placed in 70% EtOH until paraffin embedding. The paraffin embedding was performed as follows. The cornea was sequentially placed in 96% EtOH for 2 × 15 min and in 99% EtOH for 2 × 15 min, washed in Tissue-Clear (Sakura Finetek Europe, Zoeterwoude, NL, USA) for 2 × 30 min, soaked in melted paraffin for 3 × 30 min at 58 °C and cut into two halves before being embedded in paraffin, standing up with the flat side down. The embedded cornea was cut into 5-μm-thick cross-sections, using a sliding microtome (Slide 2002 compact; pfm Produkte für die Medizin AG, Köln, Germany) and placed on glass slides. The slides with the slices were finally incubated at 58 °C for 1 hr.

### Deparaffinization and immunohistochemistry

Glass slides with cornea slices were deparaffinized in Tissue-Clear (Sakura Finetek Europe) for 2 × 10 min at r.t. followed by dehydration. The slides were washed for 5 min in dH_2_O and for 2 × 5 min in PBS at r.t., treated with proteinase K (50 μg/ml) for 10 min at r.t. and washed with PBS. For blocking, 5% goat serum was used (30 min). The slides were incubated with primary antibody overnight at +4 °C. The antibodies used were CK3 [Mouse monoclonal, AE5 (ab77869); Abcam, Cambridge, UK), a marker for corneal epithelial cells, CK15 [Rabbit monoclonal, EPR1614Y (ab52816); Abcam), a marker for corneal epithelial progenitor cells, CK19 [Mouse monoclonal, BA-17 [ab7755]; Abcam), a marker for conjunctival epithelial cells, PAX6 [Mouse monoclonal, AD2.38 (ab78545), Abcam], a marker for a transcription factor important in the development of the eye and hES-Cellect (hES-Cellect™; Cellartis AB, Göteborg, Sweden), a monoclonal antibody specific for pluripotent hESCs. Slides were washed in PBS and incubated with a secondary antibody (Alexa Fluor goat anti-mouse or anti-rabbit ab; Invitrogen, Eugene, OR, USA) for 3–5 hr at r.t. and in the dark, washed once more in PBS and mounted in Vectashield with DAPI (Vector Laboratories, Inc, Burlingame, CA, USA). The analysis was performed on a Nikon fluorescence microscope equipped with DAPI, TRITC and FITC filters (360, 490 and 570 nm).

This study was conducted in accordance with the tenets of the Declaration of Helsinki and with permission from the Ethical Committee of the University of Gothenburg.

## Results

### Time-lapse

In the time-lapse system, we were able to record the outgrowth of cells from two HESC grafts through time-lapse. The course of events can be summarized as follows: 10 mins after transplanting the cell pieces onto the Bowman’s membrane, the cell sheets decreased dramatically in size forming a multilayered cell clump. Within the next 12–18 hr, the transplanted cell pieces attached to the surface of the corneal tissue and began to grow. During this period, the epithelial cells located at the wound edges began to move (sliding) to close the exposed area, as previously described (Hardarson et al. 2004). As the two cell types met, the epithelial cells ‘streamed’ on both sides of the transplanted cell pieces, completely surrounding them after several hours (10–15 hr). The transplanted cells continued to grow in an apparently monocelllayer, and as the experiment proceeded (see Video clip S1), it became more and more difficult to distinguish between the transplanted cell population and the recipient cell population, because they did not differ much in their gross morphology. We were able to detect the presence and the amount of transplanted cells by short exposure of UV light to detect their expressed GFP. From these images which were later confirmed immunohistochemically, it was apparent that the outgrowing hES-like cells did not mix with the epithelial cells but rather pushed them aside as they grew.

### Immunohistochemistry

**Fig. 1:**
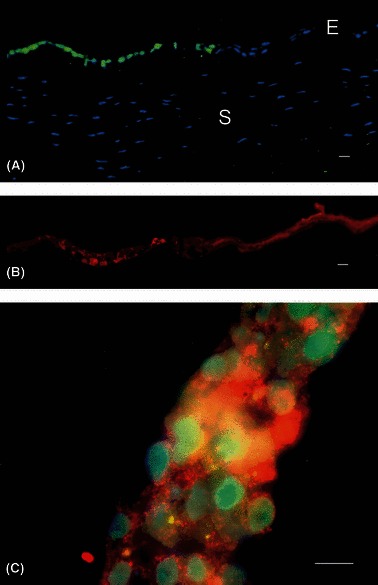
(A) A composition of photographs taken with DAPI filter (blue) and FITC filter (green), showing a part of a cross-section of a corneal button with the border between recipient corneal epithelial cells (blue nuclei) and transplanted human embryonic stem cells (hESCs) (green). The nuclei of the stromal (S) cells are seen as blue elongated structures below the epithelial (E) layer (bar 20 μm). (B) The same section as in (A) showing the expression of CK3 only (TRITC filter). Both recipient epithelial cells and grafted cells are expressing CK3 (red) (bar 20 μm). (C) Merged image of photographs taken with DAPI, FITC and TRITC filters, showing a four cell layer of transplanted hESCs (green), situated centrally of the same graft as in (A) and (B) expressing CK3 (red) (bar 10 μm).

The GFP signal remained strong throughout the preparations allowing us to easily find the regions of interest and to distinguish between the transplanted and recipient cells. All sections containing hESCs were analysed as revealed in Fig 1A, there was a distinct border where the two cell populations met and we could not see any mixing between the two populations of cells. The hESC-derived cells needed a preculture period of at least 16 days in continuous culture before transplantation (4, 7–10, 16 and 22 days in preculture were tested), to allow them to differentiate into cells expressing corneal epithelial markers (PAX6 and CK3) during culture on the cornea. Using a preculture of 4–10 days only, resulted in the expression of PAX6 but not CK3. These hESCs also showed a weak expression of the stem cell marker hES-Cellect in some sections. At the time of transplantation, the precultured hESC-derived cells were negative for PAX6, CK3, CK15 and CK19 and needed 3 days in culture on Bowman′s membrane to initiate the expression of the cornea-related marker PAX6 and 6 days to express also CK3 (2–4 and 6–9 days in culture on Bowman′s membrane were tested). The expression of CK3 (Fig. 1B) was higher in the transplanted cells closest to the recipient epithelial cells than in the more centrally localized cells. Human embryonic stem cells expressing both PAX6 and CK3 lost their expression of the pluripotent stem cell marker hES-Cellect.

## Discussion

Ahmad et al. (2007) successfully cultured hESCs on culture plates coated with collagen IV, laminin and fibronectin, using medium conditioned by human limbal fibroblasts; the result was limbal epithelial-like cells expressing CK3 and CK12. Homma et al. (2004) induced mouse embryonic stem cells to differentiate into corneal epithelial-like cells after culturing the cells on type IV collagen. The differentiated cells were successfully transplanted onto damaged mouse corneas. In a follow-up study, Kumagi et al. (2010) let *cynomolgus* monkey embryonic stem cells, treated in a similar way to the mouse ES cells, differentiate into epithelial-like cells. These cells were transplanted to injured mouse cornea, where they formed multiple cell layers.

In this study, we have shown that it is possible to transplant precultured cells derived from hESCs onto exposed (partly stripped from epithelial cells) Bowman’s membrane of the human cornea. The transplanted cells attached efficiently to the membrane and were not removed by the sliding forces from the recipient epithelial cells during wound healing. Furthermore, 1–4 cell layers of epithelial-like cells were formed by the transplanted cells (Fig. 1A,C). The transplanted cells grew out from the multilayered graft in a monocelllayer towards the proliferating front of epithelial cells originating from the damaged cornea. As the two cell populations meet, no mixing of host and transplanted cells can be found but the two cell populations are in close contact with each other as shown in Fig. 1A. Here, we have demonstrated that provided that the hESCs are precultured for at least 16 days in differentiation medium and then co-cultured with human corneal buttons 7.5–8 mm in size (without limbus) denuded centrally of epithelium, but with some remaining peripheral epithelium for at least 6 days, the transplanted cells will initiate the expression of relevant corneal epithelial markers such as CK3 and PAX6. The PAX6-/CK3-positive transplanted cells lost their expression of the pluripotent stem cell marker hES-Cellect. This study shows that it is possible to transplant hESC-derived and precultured cells directly onto Bowman’s membrane with the epithelial layer partially removed and to get these cells to establish, grow and differentiate into corneal epithelial-like cells *in vitro*.

## Supporting Information

**Video Clip S1.** A time-lapse sequence showing a piece of human embryonic stem cells growing on an area that has been stripped clean of epithelial cells.

As a service to our authors and readers, this journal provides supporting information supplied by the authors. Such materials are peer-reviewed and may be re-organized for online delivery, but are not copy-edited. Technical support issues arising from supporting information (other than missing files) should be addressed to the authors.
